# Physical Harm and Death in the Context of Coercive Measures in Psychiatric Patients: A Systematic Review

**DOI:** 10.3389/fpsyt.2019.00400

**Published:** 2019-06-11

**Authors:** Xenia A. K. Kersting, Sophie Hirsch, Tilman Steinert

**Affiliations:** ^1^Clinic for Psychiatry and Psychotherapy, University Hospital Bonn, Bonn, Germany; ^2^MVZ Venusberg of the University Hospital Bonn, Bonn, Germany; ^3^Clinic for Psychiatry and Psychotherapy I, Ulm University (Weissenau), Ulm, Germany; ^4^Zentrum für Psychiatrie Suedwuerttemberg, Weissenau, Germany

**Keywords:** coercion, harm, side-effect, seclusion, restraint

## Abstract

**Background:** For centuries coercive measures in psychiatry have been means of averting acute danger. It has been known for almost as long that these measures can lead to harm or even death to those affected. Over the past two decades the topic has increasingly been the subject of scientific discussion and research. While the legal and ethical preconditions for coercive measures in psychiatry as well as epidemiological studies on their incidence and patients’ subjective experiences have increasingly come into focus, research on possible adverse events has lagged behind. To our knowledge there is no systematic review on the harmful or even fatal physical adverse effects of coercive interventions in psychiatry.

**Methods:** We searched the databases PubMed and CINAHL for primary literature with a search string based on the PICO framework including key words describing different psychiatric diagnoses, coercive measures, and harms.

**Results:** In total, 67 eligible studies (mainly case reports and case series) of very heterogeneous quality were included. Two RCTs were found reporting position-dependent cardiac deterioration, but were, however, carried out with healthy people and were characterized by a small number of cases. Death was the most frequently reported harm: cardiac arrest by chest compression in 14 studies, cardiac arrest by strangulation in 9, and pulmonary embolism in 8 studies. Further harms were, among others, venous thromboembolism and injuries. Injuries during physical restraint were reported in 0.8–4% of cases. For other kinds of coercive interventions, there are no sufficient data. Venous thromboembolism occurred in a considerable percentage of cases during mechanical restraint, also under prophylaxis. The most commonly reported coercive measure was restraint, distinguishing in mechanical restraint (43 studies), physical restraint (22 studies), bedrails (eight studies), vest restraint (7 studies), and chair restraint (6 studies). Forced medication was explicitly mentioned only in two, but seems to have occurred in nine studies. Six studies included seclusion.

**Conclusion:** Coercive measures can lead to physical harm or even death. However, there is a significant lack of data on the incidence of such adverse events related to coercive interventions. Though reported anecdotally, physical adverse events during seclusion appear to be highly underresearched.

## Introduction

### Background

Coercive measures have been in use at least since the beginning of written records on mental illnesses and their treatment. While nowadays the primary reason for their use is prevention of danger, when aggressive or violent behavior against self or others cannot be controlled otherwise, in former times coercion was considered as treatment in itself or even used as punishment.

In ancient times, Celsus already advised protecting the patient from harm by binding him and recommended causing fear and fright as well as inflicting pain (1). In the late Middle Ages as well as in the modern age, the mentally ill were chained and locked away, and pieces of equipment were developed for treatment that rather reminds of torture methods (2).

Essentially, coercive measures can be subdivided into coercive treatment (usually with drugs, but also in rare cases by electroconvulsive therapy), and “chemical restraint” (sedative medication, with flowing transitions between treatment and restraint) on the one hand, and freedom-restricting measures such as seclusion and restraint on the other (3).

The fact that coercive measures used to prevent harm can have dangerous or even fatal consequences has been a well-known fact since the beginning of psychiatric institutions and has been controversially discussed (4, 5). But not until the last two decades have coercive measures increasingly become the subject of scientific investigations.

The “White Paper on the Protection of Human Rights and Dignity of People Suffering from Mental Disorders, especially those placed as invalids in a psychiatric establishment” (6), the ratification of the United Nations Convention on the Rights of Persons with Disabilities (7), and the German guideline “Therapeutic measures for aggressive behavior in psychiatry and psychotherapy” (8), updated in 2018 (“Prevention of coercion: prevention and therapy of aggressive behavior in adults”) mark important steps to establish a framework for the use of coercive measures in psychiatric institutions and general hospitals, based on ethics, law, and evidence.

While the legal and ethical conditions of coercive measures in psychiatry have been highlighted extensively and epidemiological studies of their incidence have become increasingly available, research on adverse effects and complications has lagged behind. Adverse effects encompass traumatic experiences and psychological sequels in a wider sense (9) as well as harmful and even fatal physical effects. The latter have repeatedly been the subject of case reports and are well known to clinicians. However, to our knowledge, there has been no systematic review so far.

### Objectives

The systematic review sets out to i) identify all kinds of reported physical harm due to the use of coercive interventions on the mentally ill and ii) estimate expected frequencies of these adverse events depending on the use of different measures.

## Methods

The databases PubMed and Cumulative Index to Nursing & Allied Health Literature (CINAHL) were systematically searched for publications that present data on harm due to coercive measures in adult psychiatric patients according to the Preferred Reporting Items for Systematic Reviews and Meta-Analyses (PRISMA) recommendations (10). In accordance with the Patient-Intervention-Comparison-Control (PICO) framework we combined, by using Boolean operators, the keywords related to the following descriptors (**Table 1**): “Person” (9 key words), “Intervention” (10 key words), and “Outcome” (20 key words). The key words were truncated and provided with wildcard characters after the word stem to identify similar words and completed with MeSH-Ters for the search in PubMed.

**Table 1:** Generation of the search string: Keywords, which serve the descriptors of the PICO framework, were separated with “OR” for the search, the respective columns of the descriptors were connected with “AND”. In PubMed the search string was completed with the following MeSH-terms: “affective psychosis, bipolar”; “behavior disorder, disruptive”; “impulse control disorders”; “mood disorders”; “neurocognitive disorders”; “neurodevelopmental disorders”; “personality disorders”; “paranoid disorders”; “psychotic disorders”; “schizophrenia”; “posttraumatic stress disorder” were added to the descriptor “person”, “restraint, physical”; “coercion” were added to the descriptor “intervention” and “death”; “asphyxia”; “mortality”; “fatal outcome”; “patient safety”; “patient harm”; “safety management”; “psychology”; “adverse effects” were added to the descriptor “outcome”.

Descriptor 1 = person	Descriptor 2 = intervention	Descriptor 3 = outcome
Mental*	Restrain*	Dead*
psychiatr*	seclu*	Death*
schizo*	coerci*	Letal*
autis*	Containment	Fatal*
delir*	compulsor*	Harm
dement*	involuntar*	“Side effect*”
Intellect*	*Forced	“Adverse effect”
brain injur*	Detained	Accident*
Bipolar	Commitment	injur*
	“Prone position”	complicat*
		Risk*
		“Patient safety”
		CIRS
		“Critical incident report system*”
		mortalit*
		“Standardized mortality rat*”
		SMR*
		asphyx*
		“commotio cordis*”
		Rhabdomyolysis

The search string was used to search the existing titles and abstracts. The search encompassed all articles published by September 3, 2018, with an open beginning. All articles that were found with the English search string were included, if they met the previously determined inclusion criteria and could be translated. We included articles that presented data on physical harm possibly caused by coercive measures in adults with a psychiatric diagnosis, except for mental and development retardation (ICD-10 blocks F7x and F8x). These diagnoses were excluded because of the partly alternate objectives of coercive measures in this particular context, e.g. operant learning (11). We did not constrain the search to psychiatric settings and thus also included measures taken against people with mental illness by the police. Excluding such articles would have resulted in a loss of knowledge about possible harm mechanisms in compulsory measures. We also included two studies reporting on coercive measures involving healthy subjects who could be considered as experimental persons simulating to suffer from mental illness.

Articles that did not report own data (e.g., reviews) were excluded, as well as articles solely documenting psychological harm. Nevertheless, psychological harm is a very important aspect, but was not the objective of this study.

The first author, XK, performed the initial screening of title and abstract of the initially found articles. The final eligibility of studies was determined after full text screening and evaluation and discussion between XK and TS.

The included articles were then analyzed with regard to their methodology, the investigated harm, the applied coercive measures, and the diagnoses of the affected persons. The results were classified by content and then evaluated. If sufficient studies were to be found meta-analyses would be performed.

Specifically, the studies were classified as follows:

1. Case reports: reports on individual cases independent of a group observation
2. Case series: several similar but independent cases reported together
3. Association studies: studies not primarily investigating groups of patients who were subjected to coercive interventions but using coercion as a predictor for harm, when parts of the group were affected by these interventions
4. Epidemiological studies: studies including a population that experienced coercive measures and investigating the occurrence of adverse events
5. Case-control studies: studies comparing the effect of a coercive measure in a group with a control group not affected by such measure
6. Experimental studies and randomized controlled studies: studies investigating measures to groups randomized in advance

Afterwards, the studies were assigned to clinical syndromes, oriented on the International Classification of Diseases 10th Revision (ICD-10), as several of the studies did not describe their population according to the fixed diagnostic criteria of the ICD-10, and grouped as follows:

1. Syndromes of dementia (F00-F03)
2. “Excited delirium” (accepted diagnosis in the US, not existent in ICD-10) and states of excitation
3. Delirium not caused by alcohol or drug abuse (F05)
4. Psychoactive substance use (F1x)
5. Schizophrenia and psychotic disorders (F2x)
6. Manic episodes were summarized together with the much rarer depressive episodes under affective disorders (F3x)
7. Personality disorders
8. Others: psychiatric disorders from chapter 4 of the ICD-10 (anxiety, dissociative, stress-related, somatoform, and other non-psychotic mental disorders) were subsumed to “others,” because of their rarity among the included studies, also the behavioral syndromes associated with physiological disturbances and physical factors that did not appear in our search (F5x), and studies that (partially) did not name the diagnosis in individual cases or summarized larger cohorts under the term “further diagnoses” without describing conspicuous behavior or symptoms in detail
9. Two studies that conducted investigations on healthy subjects

The investigated coercive measures were classified as follows (12, 13):

1. Restraint, which was further divided into
  - physical restraint, meaning immobilizing a patient by holding him manually
  - mechanical restraint, which usually means the use of belts to fix a patient to a bed, mostly four-point or five-point (but also one-point to 11-point)
  - mechanical restraint by chair restraint
  - mechanical restraint by bed rails
  - vest restraint
2. Seclusion, meaning separating the patient in a locked room
3. Forced medication, meaning oral or parenteral (intravenous or intramuscular) application of medication by force or by definite psychological pressure, e.g., announcing forced parenteral medication if medication is not immediately taken orally
4. Studies reporting the additional use of arms in police custody were classified separately, because it can be assumed that other harm mechanisms influence the outcome

## Results

### Search Query Results and General Characteristics of the Studies

**Figure 1:**
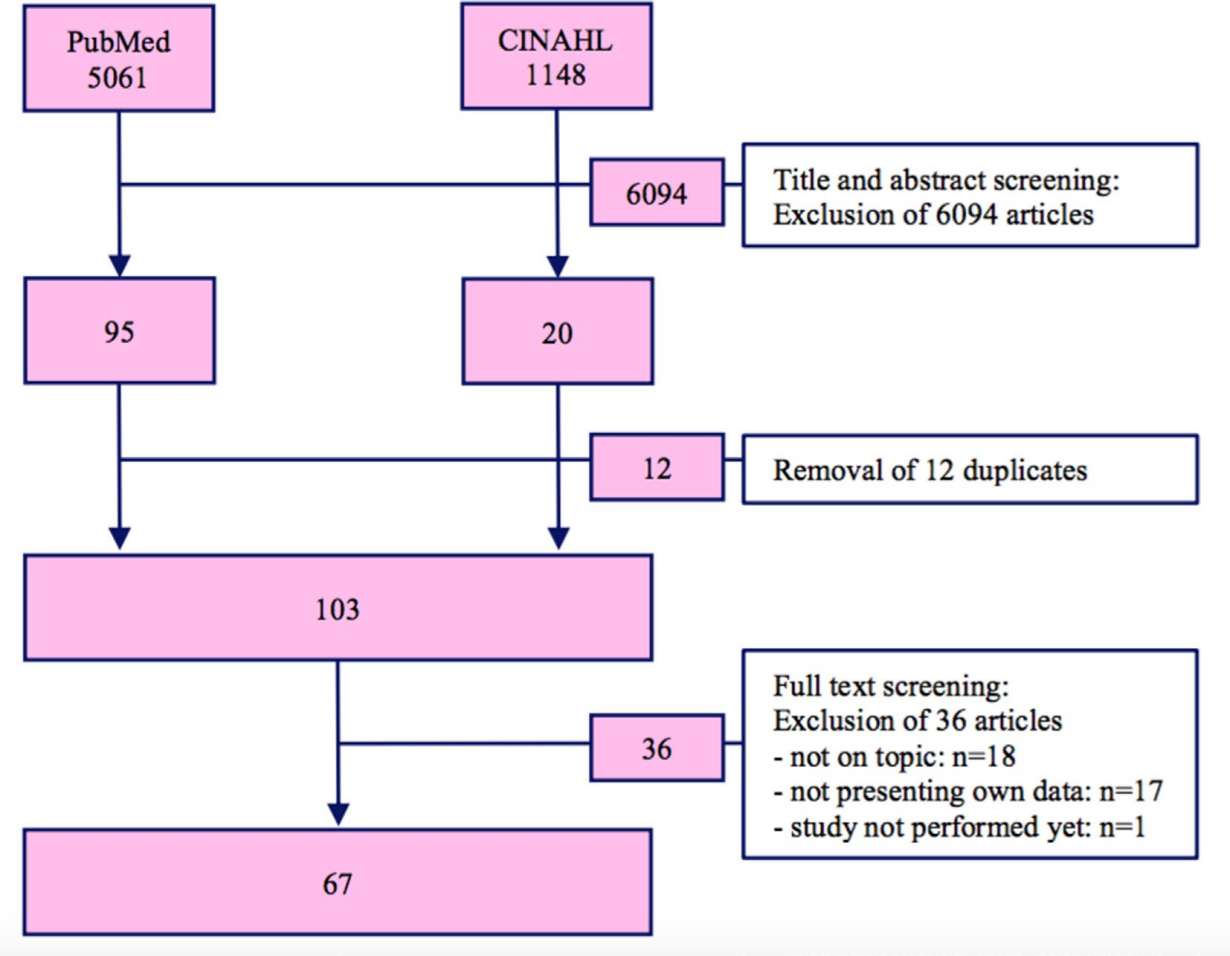
Flowchart of the study inclusion.

The initial search yielded 6,209 hits, of which 6,096 were excluded during the initial screening. From the remaining 113 articles 12 were excluded due to being duplicates and further 36 articles were excluded after full text screening for the following reasons: off topic [not a psychiatric patient (one study) or treatment (one study), mental retardation (two studies), no coercive measures (five studies), no documented somatic harm (nine studies)], not presenting own collected data (17 studies), and study not yet performed (one study); 67 could be included for the systematic review. **Figure 1** shows the inclusion process in a flow chart.

Characteristics of the included papers are displayed in **Tables 2**–**7**. The first was published in 1964, the larger part in the last two decades.

**Table 2:** Characteristics of the RCTs.

References	Content	Country, setting	Diagnosis								Coercive measure								Harm	N
			Del	Dem	ED	F1	F2	F3	F6	O.	PR	MR	CR	BR	V	S	FM	A		
Parkes (14)	Healthy subjects were investigated after exercise comparing relaxed sitting, PR in prone, and in supine position. Cardiac recovery was delayed in prone position.	UK								*	x								Delayed cardiac recovery	13
Roeggla et al. (15)	Healthy subjects were investigated in MR in prone position versus MR in upright position, prone position led to dramatic impairment of hemodynamics and respiration.	Austria								*		x							Cardiac detoriation	6

N, number of patients sustaining coercive measures; UK, United Kingdom; Del, delirium; Dem, dementia; ED, excited delirium and states of excitation; F1, mental and behavioral disorders due to psychoactive substance use; F2, schizophrenia and other psychotic disorders; F3, affective disorders; F6, personality disorders; O., other psychic or somatic diagnoses, unspecified or not labeled diagnoses; PR, physical restraint; MR, mechanical restraint; CR, chair restraint; BR, bed rails; V, restraint vests; S, seclusion; FM, forced medication; A, use of arms; *, healthy subjects.

**Table 3:** Characteristics of the case control study.

References	Content	Country, setting	Diagnosis								Coercive measure								Harm	N
			Del	Dem	ED	F1	F2	F3	F6	O.	PR	MR	CR	BR	V	S	FM	A		
Hatta et al. (16)	The patients (diagnoses not mentioned) with mechanical restraints (106) in a hospital were compared to those without restraints (528). MR increases the risk of DILI even with the same medication.	Japan, psych. clinic								x		x							DILI	106

N, number of patients sustaining coercive measures; psych., psychiatric; DILI, drug induced liver injury; Del, delirium; Dem, dementia; ED, excited delirium and states of excitation; F1, mental and behavioral disorders due to psychoactive substance use; F2, schizophrenia and other psychotic disorders; F3, affective disorders; F6, personality disorders; O., other psychic or somatic diagnoses, unspecified or not labeled diagnoses; PR, physical restraint; MR, mechanical restraint; CR, chair restraint; BR, bed rails; V, restraint vests; S, seclusion; FM, forced medication; A, use of arms.

**Table 4:** Characteristics of the epidemiological studies.

References	Content	Country, setting	Diagnosis								Coercive measure								Harm	N
			Del	Dem	ED	F1	F2	F3	F6	O.	PR	MR	CR	BR	V	S	FM	A		
Hall et al. (17)	Investigation of 3,564 cases with mental illness in police custody (PR), comparing prone and supine position, no negative effects in prone, one death in supine.	Canada, police custody			x	x					x							x	Death	3,564
De Hert et al. (18)	Analyzing the data of all patients with neuroleptic treatment, of whom 170 were secluded and 138 secluded and restraint, with regard to the occurrence of DVT. No case of DVT occurred, preventive measures in 38%.	Belgium, psych. clinic					x					x				x	x		DVT (no case)	170 and 138
Strote et al. (19)	From all 66 cases with ED in police custody of 3 years, 65% were brought to the emergency department, 9% of those (6% of all) had injuries.	USA, police custody			x						x	x						x	Injuries	66
Ford (20)	Data from 2013 to 2017 collected by the Liberal Democratics *via* a Freedom of Information Act Request, injury rate by physical restraint was 1,05% in patients (3% in staff).	UK, patients from Mental Health Trust								x	x								Injuries	216,018
Lancaster et al. (21)	All cases (260 patients, 680 events) of physical restraint were analyzed regarding to the position. Injury rate across incidents 4% in patients (17% in staff).	UK, psych. clinic								x	x								Injuries	260
Ishida et al. (22)	All of the 190 patients with mechanical restraints were screened for DVT in several steps. Despite of prophylaxis 11.6% developed DVT. Duration of restraint (as well as medication and somatic diseases) were significantly correlated.	Japan, psych. clinic	x	x		x	x	x	x	x		x							DVT	181
Stubbs et al. (23)	Analysis of injuries (14 in 11 patients) after physical restraint (1,427 events in 75 patients). Patient injury rate 14.7%.	UK, rehabilitation clinic								x	x								Injuries	75
Pinninti et al. (24)	Letter to the editor, report of all (1,403) mechanical restraints, and all patient deaths (four, all without restraints) in 5 years with an average of 950 commitments per year, average annual rate of 4.6 restraints per 1,000 patient-days, death rate in restraint 0%.	USA, psych. clinic								x		x							Death (no case)	1,403
Mattson et al. (25)	63 patients in seclusion were compared (not matched) to 160 non secluded patients. Different harm was documented in 33 of the secluded, above all the oversight of complications. Eight showed self-injury, three showed physical deterioration. A comparison of the harm between the two groups did not take place.	USA, psych. clinic					x	x	x	x						x			Injuries, self-harm, oversight of complications	63
Lofgren et al. (26)	Over 13 weeks prospective all patients with mechanical restraints were included and harm was recorded. Restraints led to an increased mortality, restraints longer than 4 days led to increased infections, incontinence, pressure sores (no control group but dose-effect).	USA, general hospital		x								x			x				Nosocomial infections, pressure sores, increased mortality, incontinence	102
Nielssen et al. (27)	40 randomly selected patient files of involuntarily committed patients from 18 psych. hospitals each were investigated in regard to the application of intravenous medication (132 patients, 27%) and possible harm: dystonia (49 cases, 37%), hypotension (11 cases, 8%), confusion (seven cases, 5%), phlebitis (three cases, 2%).	Australia, psych. clinic				x	x	x	x	x							x		Dystonia, hypotension, confusion	132

N, number of patients sustaining coercive measures; UK, United Kingdom; USA, United States of America; psych., psychiatric; Del, delirium; Dem, dementia; ED, excited delirium and states of excitation; F1, mental and behavioral disorders due to psychoactive substance use; F2, schizophrenia and other psychotic disorders; F3, affective disorders; F6, personality disorders; O., other psychic or somatic diagnoses, unspecified or not labeled diagnoses; PR, physical restraint; MR, mechanical restraint; CR, chair restraint; BR, bed rails; V, restraint vests; S, seclusion; FM, forced medication; A, use of arms; DVT, deep vein thrombosis.

**Table 5:** Characteristics of the association studies.

References	Content	Country, setting	Diagnosis								Coercive measure								Harm	N
			Del	Dem	ED	F1	F2	F3	F6	O.	PR	MR	CR	BR	V	S	FM	A		
Grover et al. (28)	Prospective evaluation of all patients with delirium. 49 were restrained. Risk factors for delirium and increased mortality were younger age, alcohol, and the use of restraints.	India, general hospital	x									x							Increased mortality	49
Bredthauer et al. (29)	All patients were analyzed regarding restraints (37 cases), risk factors, and the incidence of falls. Falls were equally often in restrained and unrestrained patients, fractures more often with restraints.	Germany, psych. clinic	x	x		x	x	x		x		x	x	x					Fractures, falls	37
Fonad et al. (30)	Aggregated data, investigation of falls, and fall risks. Correlations between falls and the use of restraints remain unclear.	Sweden, dementia and somatic ward		x						x		x	x	x					Falls	?
Honkonen et al. (31)	Investigation of the mortality of inpatients. 10% of all died within the 2-year-follow up (rate explained by many severe alcohol addicted). Use of restraints led to an increased mortality, but a direct causality was not assumed. 67 of 424 patients with restraints died (16%).	Finland, psych. clinic				x	x	x		x	x	x				x	x		Death, increased mortality	424
Windfuhr et al. (32)	283 cases of SUD between 3/99 and 2005 were matched with other patients, nine deaths had sustained mechanical restraint and/or seclusion. Twice as many died after MR/S as in the controls, which was not significant.	UK, psych. Clinic		x			x	x				x				x			Death (SUD)	9
Michaud (33)	Analysis of a forensic database of all restraint related deaths in police custody.	Canada, police custody			x						x	x						x	Death	14
Robbins et al. (34)	Patients of a general hospital were in course investigated regarding MR (in 37 cases = 17%). The patients with restraints were more likely to die but also more seriously ill, so a causality for the mortality and more nosocomial infections was not assumed. Minor skin lesions were documented.	USA, general hospital		x								x	x		x				Increased mortality, minor skin lesions, nosocomial infections	37
Mion et al. (35)	Of all patients, those who fall were compared to those who did not. 61% of the fallers had received restraints versus 22% of the non fallers.	USA, rehabilitation clinic								x		x							Falls	49
Dharmarajan et al. (36)	Data from the “Project Recovery” was analyzed, restraints are associated with increased mortality, a causality could not be assumed.	USA, general hospital	x									x							Increased mortality	17
Arbesman et al. (37)	Patients who fall were compared to those that did not fall (same duration of stay). Restraints double the fall risk, 25% of the fallers and 8% of the non fallers had recieved restraints. A significance is assumed only for longer durations of the restraints.	USA, hospital with general ward and psych. ward								x		x							Falls	83
Gaertner et al. (38)	Episodes of VTE were identified from a database. The clinical, somatic, psychiatric, and therapeutic characteristics of each patient were compared with a matched control group without VTE. Restriction of mobility was equally prescribed for the patients in both groups: 12 patients (36%) in the case group and 21 patients (26%) in the control group. Continuous physical restraint was prescribed more in the case group (three (9%) versus zero patients) but this difference was not significant. Not more restriction of mobility by PR or seclusion in the case group (continuous or sequential).	France, psych. clinic	x	x		x	x	x	x	x		x				x			VTE (not increased in restraints)	33
Takeshima et al. (39)	All patients were screened for VTE in several steps, then circumstances were analyzed. VTE was observed in 2.3% (39/1,681) of all patients, in 61.1% (11/18) of catatonic patients, 4.1% (11/270) of noncatatonic restrained patients, and 1.2% (17/1,393) of non-catatonic not restrained patients.	Japan, psych. clinic					x	x		x		x							VTE	288

N, number of patients sustaining coercive measures; UK, United Kingdom; USA, United States of America; psych., psychiatric; Del, delirium; Dem, dementia; ED, excited delirium and states of excitation; F1, mental and behavioral disorders due to psychoactive substance use; F2, schizophrenia and other psychotic disorders; F3, affective disorders; F6, personality disorders; O., other psychic or somatic diagnoses, unspecified or not labeled diagnoses; PR, physical restraint; MR, mechanical restraint; CR, chair restraint; BR, bed rails; V, restraint vests; S, seclusion; FM, forced medication; A, use of arms; VTE, venous thromboembolism; SUD, sudden unexplained death.

**Table 6:** Characteristics of the case series.

References	Content	Country, setting	Diagnosis								Coercive measure								Harm	N
			Del	Dem	ED	F1	F2	F3	F6	O.	PR	MR	CR	BR	V	S	FM	A		
Karger et al. (40)	Strangulation by restraints in seven elderly people, autopsy reports.	Germany, surgical clinic, at home, nursing homes	x	x			x					x							Death	7
Hem et al. (41)	Two cases of PE after mechanical restraint, one died.	Norway, psych. clinic					x	x				x							DVT, PE, death from PE	2
Stefanović et al. (42)	Autopsy report of five deaths from PE after mechanical restraints.	Serbia, psych. clinic					x					x					x		Death from PE	5
Pötsch et al. (43)	Five deaths due to strangulation/asphyxiation by mechanical restraints (restraint systems in bed, CR, BR) in elderly people in nursing homes.	Germany, nursing home	x	x			x			x		x	x	x					Death	5
O’Halloran et al. (44)	11 cases of death in police custody (physical restraint in prone position; in three cases arms (Taser, batons) were used).	California, police custody			x	x	x				x	x						x	Death	11
Mohsenian et al. (45)	Six cases of asphyxia due to strangulation by mechanical restraints and bed rails (diagnosis of case 4 is unknown).	Germany, psych. clinic, general hospital, nursing home	x	x						x		x		x					Death	6
Fariña-López et al. (46)	Three cases of death by strangulation in elderly with dementia restrained by abdominal belt and BR.	Spain, psych. clinic, nursing home		x								x		x					Death	3
Pollanen et al. (47)	Investigation of 21 cases with ED that died in police custody with physical restraint.	Canada, police custody			x						x							x	Death by heart failure	21
Stratton et al. (48)	Investigation of 18 cases with ED that died from cardiapulmonary arrest in police custody with physical an mechanical restraints.	Canada, police custody			x						x	x						x	Death	18
Dickson et al. (49)	Three cases of death from PE after mechanical restraint.	Canada, psych. clinic		x				x				x							Death from PE	3
Stratton et al. (50)	Two cases of cardiopulmonary arrest after physical restraint and hobble restraint with handcuffs in police custody.	Canada, police custody			x						x	x							Death	2
Lazarus (51)	Two cases of death from PE during mechanical restraint (8 days and 1 day duration).	USA, psych. clinic					x	x				x							Death from PE, DVT	2
Pedal et al. (52)	Four deaths of physical restraint in police custody.	Germany, police custody			x						x								Death	4
Miles et al. (53)	122 cases of deaths as a direct consequence of mechanical restraints were aggregated from different databases, for at least one out of 1,000 deaths in nursing homes MR shall be causative.	USA, general hospitals, nursing homes		x								x	x	x					Death	122
Mirchandani et al. (54)	Five cases of death in police custody with PR and use of arms were analyzed (all sustained head injuries that were not causal for death).	USA, police custody			x	x					x							x	Death	5
Krexi et al. (55)	Analysis of a cardiologic database, 34 cases of sudden cardiac death after struggling against physical restraint.	UK, psych. clinic, at home, police custody					x			x	x								Death by heart failure	34
Bell et al. (56)	Two cases from a database died from strangulation in restraint vests and chairs (the other cases did not sustain coercive measures).	USA		x									x		x				Death by strangulation	2
Hammer et al. (57)	Two cases of death by strangulation in restraint vests.	Germany, psych. clinic					x								x				Death by strangulation	2
McArdle et al. (58)	Two cases of pneumomediastinum caused by Valsalva maneuver due to MR in police custody, no direct traumatic mechanism.	Australia, general hospital, police custody				x	x				x							x	Pneumo-mediastinum	2
Uemura et al. (59)	Autopsy reports of two cases with ED with sudden cardiac death by restraints in police custody.	Japan, psych. Clinic, police custody			x		x				x	x							Death by heart failure	4

N, number of patients sustaining coercive measures; UK, United Kingdom; USA, United States of America; psych., psychiatric; Del, delirium; Dem, dementia; ED, excited delirium and states of excitation; F1, mental and behavioral disorders due to psychoactive substance use; F2, schizophrenia and other psychotic disorders; F3, affective disorders; F6, personality disorders; O., other psychic or somatic diagnoses, unspecified or not labeled diagnoses; PR, physical restraint; MR, mechanical restraint; CR, chair restraint; BR, bed rails; V, restraint vests; S, seclusion; FM, forced medication; A, use of arms; DVT, deep vein thrombosis; PE, pulmonary embolism.

**Table 7:** Characteristics of the case reports.

References	Content	Country, setting	Diagnosis								Coercive measure								Harm	N
			Del	Dem	ED	F1	F2	F3	F6	O.	PR	MR	CR	BR	V	S	FM	A		
Nissen et al. (60)	Schizophrenic patient was resuscitated after cardipulmonary arrest in physical restraint (prone position).	Norway, psych. clinic					x				x								Death	1
Wöllner et al. (61)	Schizophrenic patient was mechanically restrained, but not monitored, because of suicidality and aggression and died after jumping out of the window.	Germany, psych. clinic					x					x							Death	1
O’Halloran (62)	Schizophrenic patient with asphyxia during physical restraint (prone position).	California, psych. clinic					x				x								Death	1
Morrison et al. (63)	Asphyxiation after 90 minutes physical restraint in prone position.	Scotland, psych. clinic				x	x				x						x		Death	1
Hewer et al. (64)	Schizophrenic patient sustained mechanical restraint and compulsive medication with olanzapine and lorazepam and died from PE.	Germany, psych. clinic					x					x					x		PE, death from PE	1
Siebert et al. (65)	Cardiorespiratory arrest after >4 minutes PR in prone position, patient with schizophrenia (case 2 was not an adult).	Florida, psych. clinic					x				x								Death	1
Schrag et al. (66)	Asphyxiation death after physical restraint in prone position (case 2 did not sustain a coercive measure).	Switzerland, police custody			x						x								Death	1
Raju et al. (67)	Death due to hypovolemic shock (hemoperitoneum) after trauma to the liver during mechanical restraint.	India, psych. clinic					x					x					x		Intraabdominal bleeding to death	1
Nielsen (68)	Restraint death by PE after 6 days in mechanical restraints.	Denmark, psych. department					x					x					x		Death from PE	1
Miles (69)	Asphyxiation during restraint by vest and bed rails.	USA		x										x	x				Death	1
Laursen et al. (70)	Schizophrenic patient survived DVT and PE after 13 days with mechanical restraints.	Denmark, psych. department					x					x							DVT, PE	1
Cecchi et al. (71)	Death from PE after 6 days in mechanical restraints.	Italy, psych. department					x					x							Death from PE	1
Hem et al. (72)	Schizophrenic patient with DVT and PE during mechanical restraint.	Norway, psych. clinic					x					x							DVT, PE	1
Langslow (73)	Asphyxiation death of a schizophrenic patient with strangulation in vest restraint.	Australia, psych. clinic			x	x						x			x				Death by strangulation	1
Leth et al. (74)	Autopsy report, one case of a schizophrenic patient with PE after 5 days in mechanical restraint.	Denmark					x					x							Death from PE	1
Robinson (75)	93 year old patient with dementia died in mechanical restraint, “collapse of will” was supposed.	Florida, psych. clinic		x								x							Death	1
Miles (76)	95 years old patient with dementia died after 4 hours struggling against (and three times escaping from) the vest restraint, 5 mg haloperidol was given intramuscular.	USA		x										x	x		x		Death by heart failure	1
Robinson et al. (77)	Heart failure after struggling against MR in a patient with dementia.	USA, general hospital		x								x							Death by heart failure	1
Nelson et al. (78)	Schizophrenic patient in seclusion died from asphyxia after crawling inside the mattress whilst unobserved.	UK, psych. clinic					x									x			Death by asphyxia	1
McLardy-Smith et al. (79)	Ischemic contracture after the application of mechanical restraints to the hands for 48 hours in a patient with mania.	UK, police custody						x				x							Contracture of the hands	1
Skowronek et al. (80)	Patient with mechanical restraints over 24 days and medication with clozapine (toxic blood levels) died by heart failure.	Poland, psych. clinic					x					x							Death by heart failure	1

N, number of patients sustaining coercive measures; UK, United Kingdom; USA, United States of America; psych., psychiatric; Del, delirium; Dem, dementia; ED, excited delirium and states of excitation; F1, mental and behavioral disorders due to psychoactive substance use; F2, schizophrenia and other psychotic disorders; F3, affective disorders; F6, personality disorders; O., other psychic or somatic diagnoses, unspecified or not labeled diagnoses; PR, physical restraint; MR, mechanical restraint; CR, chair restraint; BR, bed rails; V, restraint vests; S, seclusion; FM, forced medication; A, use of arms; DVT, deep vein thrombosis; PE, pulmonary embolism.

Analyzed with regard to their methodology, two studies were experimental RCTs that investigated coercive measures on healthy subjects (14, 15) with small sample sizes of 13 respectively six probands, 1 study was a case-control study (16), 11 studies were classified as epidemiological studies (17–27), 12 as association studies (28–39), and the majority as case series (40–59) and case reports (60–80). A systematic quality assessment of the included studies as it is usually done with RCTs being included in meta-analyses (risk of bias) and calculation of risks was not possible due to the diverse methodology of the studies, with different designs, different investigated outcomes, and partly incomplete data. In particular, when calculating the mortality as some studies did, many interacting factors are involved.

Since the epidemiological studies allow deductions to be drawn from the frequency of harm, these are listed at the start, before all the studies found are subsequently presented with regard to the harm, the coercive measures, and the diagnoses.

### Results of the Epidemiological Studies

Three studies investigating the injury rate with physical restraint (20, 21, 23) can be grouped together. Ford (20) published a calculated injury rate of 1.05% in all 216,018 patients with physical restraint, with data from 2013 to 2017 collected by 40 mental health trusts. Lancaster et al. (21) calculated an injury rate of 4% across 680 incidents of physical restraint involving 280 patients from one Mental Health Trust from 1999 to 2001. Stubbs and Alderman (23) investigated patients with brain injury in a rehabilitation setting over the time period of 1 year and calculated a patient injury rate of 14.7% (11 out of 75 patients suffered injuries in 1,427 events of physical restraint). Strote et al. (19) found a higher rate of 9%, but related to police arrests including use of weapons.

Two further epidemiological studies can be grouped together as investigating venous thromboembolism (VTE) in patients receiving mechanical restraint (and seclusion). De Hert et al. (18) did not document any case of VTE in 170 secluded patients, of which 138 were additionally mechanically restrained (176 episodes of seclusion, 196 episodes of seclusion and restraint) and 38% received VTE prophylaxis. However, Ishida et al. (22) reported an incidence of 11.6% (21 out of 181) of deep vein thrombosis in restrained patients, all of them having received prophylaxis. This difference can be partly explained by the fact that Ishida et al. may have investigated this side effect in more detail; patients with elevated D-dimers underwent Doppler ultrasounds.

Mattson and Sacks (25) investigated all 63 secluded patients from the year 1975 and documented—besides the fact that patients in seclusion received less care and complications were overseen—self-injury in 8 (12.7%) and physical deterioration in 3 patients (4.8%). Lofgren et al. (26) reported new pressure sores in 22 of the 102 (21.6%) mechanically restrained elderly patients during a 13-week period, new incontinence in 29 (28.4%), and nosocomial infections in 12 (1.8%), and also a significantly increased mortality with increasing duration of restraint (>4 days).

Nielssen et al. (27) investigated each 40 randomly selected patient files of involuntarily committed patients from 18 psychiatric hospitals with regard to the application of intravenous medication (132 patients) and possible harm: dystonia (49 cases, 37%), hypotension (11 cases, 8%), confusion (7 cases, 5%), phlebitis (3 cases, 2%).

Pinninti and Rissmiller (24) presented a hospital report of all (1,403) mechanical restraints and all patient deaths (4 deaths, all patients without restraints) within a time span of 5 years, death rate in restraint 0%. Hall et al. (17) investigated 3,564 patients in police custody over a period of 7 years, comparing physical restraint in prone and supine position and documenting no death in prone and one in supine position.

### Results of all Studies w.r.t. Type of Harm, Applied Coercive Measure, Diagnoses of the Population

#### Type of Harm

None of the studies found investigated all forms of harm in all coercive measures. In most cases, only one type of harm was examined in the context of only one or a few different coercive measures.

Death was the most frequently studied harm, documented in 42 studies (17, 24, 31–33, 40–57, 59–69, 71–78, 80) and distributed over all study types, mentioned especially in the case reports and case series. A frequent cause of death was cardiopulmonary arrest in 17 studies (17, 33, 44, 48, 50, 52, 54, 55, 59, 60, 62, 63, 65, 66, 75, 76, 80), whereby an assignment to asphyxia due to pressure on the thorax or the position (“positional asphyxia”) or heart failure usually was not mentioned by default and overlaps were common. Asphyxia caused by strangulation was mentioned in 10 studies [Refs. (40, 43, 45, 46, 53, 56, 57, 69, 73, 78), the latter one reporting a patient in seclusion crawling into the bed sheets] and pulmonary embolism in eight studies (41, 42, 49, 51, 64, 68, 71, 74). Other causes were suicide [Ref. (61), patient was mechanically restrained and inadequately monitored and jumped out of the window], bleeding to death [Ref. (67), hemoperitoneum resulting from restraints], and sudden unexplained death (32) as well as asphyxia caused by choke-hold (53) each in one study. Five studies documented an increased mortality associated with coercive measures, often without being able to deduce a direct causality (26, 28, 31, 34, 36).

The second most frequently analyzed harm was VTE in 14 studies: deep vein thrombosis of the leg (DVT) in 8 studies (18, 22, 38, 39, 41, 51, 70, 72) and pulmonary embolism (PE) in 12 studies (38, 39, 41, 42, 49, 51, 64, 68, 70–72, 74), with the consequence of death in 8 studies (41, 42, 49, 51, 64, 68, 71, 74). Gaertner et al. (38) retrospectively found out that among the analyzed patients with VTE, the restrained patients were not more frequently affected by this harm than the unrestrained. As stated above, this finding is also supported by de Hert et al. (18). In contrast to these, the other studies observed that VTE occurred even when the restrained patients were heparinized or receiving other prophylactic measures (22, 39) and the importance of regular examination and treatment of thrombosis was emphasized (22).

Harm in the form of injuries/physical traumata was reported in eight studies encompassing minor skin lesions, pressure sores, bruises, lacerations, contusions, fractures, head injuries, and not further specified injuries (19–21, 23, 26, 34, 44, 63).

Four association studies investigated and documented the correlation between the incidence of falls and the application of mechanical restraints intended to protect from falling. Nearly all showed an increased, though not significant risk of falling (29, 30, 35, 37). Notwithstanding Arbesman and Wright (37) reported a significant increase in falls, which were twice as likely when patients were restrained, attributing this to accelerated physical deterioration. Bredthauer et al. (29) as well as Mion et al. (35) could not provide significant evidence that restraint is associated with an increased risk of falling.

Nosocomial infections as a harm in the context of coercive measures as documented by Lofgren et al. and Robbins et al. (26, 34) cannot clearly be distinguished from the general frailty of those in restraints with respect to causality.

Furthermore, there are less frequently documented complications in the context of coercive measures like incontinence (26), contractures (79), and pneumomediastinum (58). Hatta et al. (16) comprehensively investigated drug-induced liver injury (DILI) in a case-control-study and found that patients with mechanical restraints were four times more at risk than patients who did not experience mechanical restraint. This result, which could potentially be attributed to the fact that patients in restraint might receive more or stronger medication, was also shown in a direct comparison of patients with identical medication. As a cause for the increased occurrence of DILI, in particular stress-associated physiological alterations were discussed.

Both randomized and controlled experimental studies reported a delayed cardiorespiratory recovery after restraint among healthy subjects (14, 15). Numerous other studies reported death in the “prone position,” e.g., Pollanen et al. (47) and Stratton et al. (48), whereas Hall et al. (17) investigated cases of prone position in police custody, and concluded that this method of restraint had no effect on the physiology of those affected.

Mattson and Sacks (25) reported self-injury during seclusion as a form of harm and additionally found out that patients in seclusion receive less attention and inadequate treatment from staff, leading to complications being overlooked.

**Figure 2:**
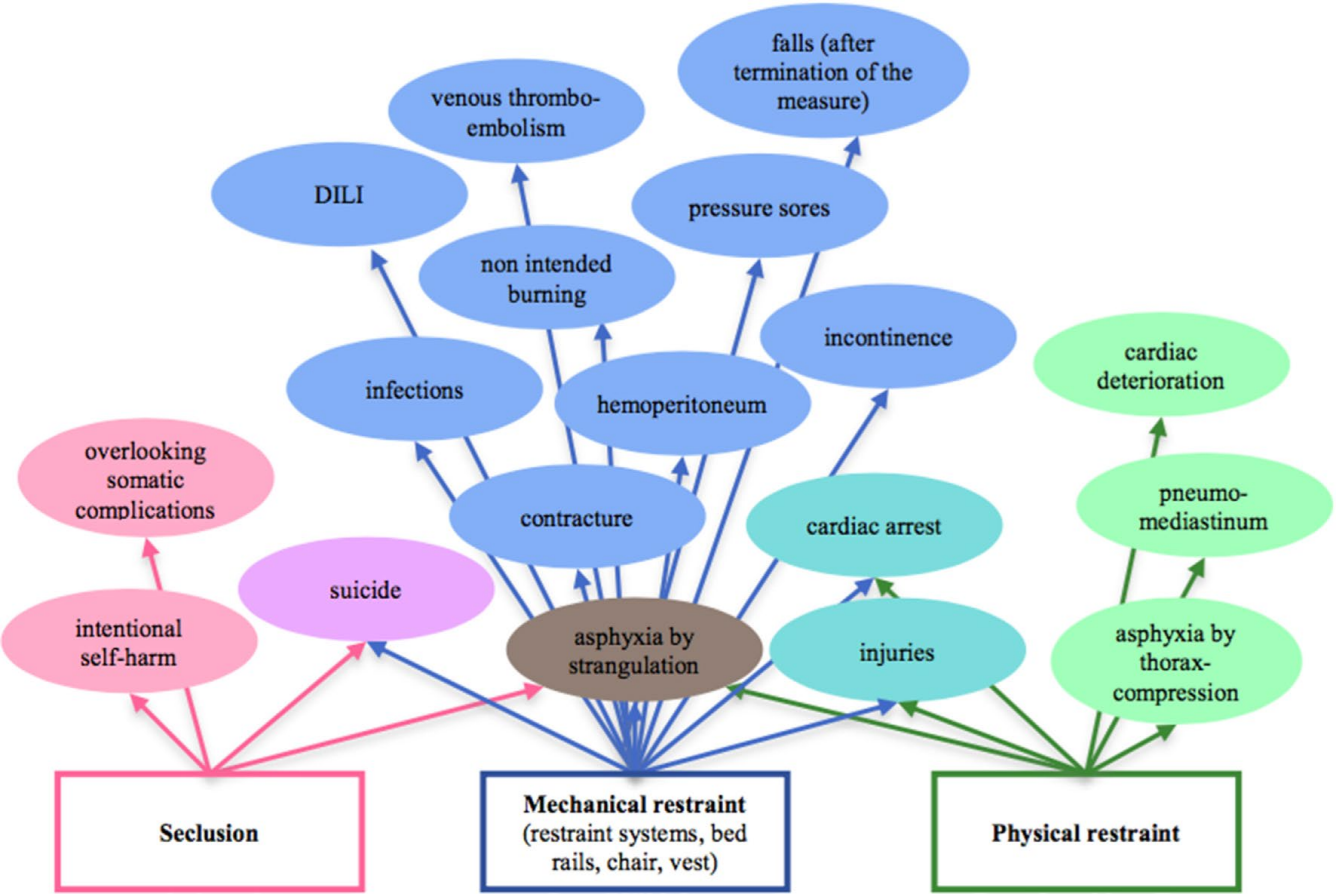
Illustrates different harms in correlation to the coercive measures physical and mechanical restraint and seclusion.

Miles and Irvine (53) presented an overview of 122 deaths in mechanical restraint, in which, among others, 4 deaths by burning are reported: three patients set the restraint on fire with the intention to escape, one person died accidently by inflamed oxygen ignited by a cigarette. **Figure 2** illustrates different harms in correlation to the different coercive measures.

A few studies did not find harm in the context of the coercive measures they investigated (17, 18, 24, 29, 35, 38).

#### Type of Coercive Measure

##### Restraint

Restraint was the most frequently reported coercive measure, subdivided into physical restraint and mechanical restraint, bed rails, chair restraint, and restraint vests. Restraint applied by police implied possible use of arms and was listed separately.

Forty-three studies reported on mechanical restraint (15, 16, 18, 19, 22, 24, 26, 28–46, 48–51, 53, 59, 61, 64, 68, 70, 71–75, 77, 80), whereby the used restraint systems varied widely. Abdominal belts, representing a one-point restraint, are mentioned as well as restraint using belts securing the patient at several points, but also measures such as binding with dressing material (34). Roeggla et al. (15) investigated a method named “hogtieing,” denoting a technique of binding hands and feet together on the back together, in healthy subjects in order to analyze physical consequences of the hogtie position.

Six studies reported chair restraint in older age (29, 39, 34, 43, 63, 56), eight studies reported bed rails (29, 30, 43, 45, 46, 53, 69, 76), and the use of restraint vests was reported in seven of the included studies (26, 34, 56, 57, 69, 73, 76). In the case of restraint vests, devices with long sleeves and leather straps on the collar were reported (57), as well as vests constructed by the manufacturer Posey, who gives clear safety instructions (81), which were disregarded in some cases (73).

Physical restraint, also named “PI” = “personal intervention,” was the subject of 22 studies (14, 17, 19–21, 23, 31, 33, 44, 47, 48, 50, 52, 54, 55, 58–60, 62, 63, 65, 66).

An important distinction relates to the body position, with comparisons between supine position and prone position. The latter has already been described as being more harmful by Reay et al. (82).

Coercive measures in police custody involve in addition to physical restraint (in prone or supine position) the special technique of hogtieing as well as the use of handcuffs as a form of mechanical restraint (17, 19, 33, 44, 47, 48, 50, 52, 54, 55, 58, 59, 66, 79), and in some cases the use of arms (17, 19, 33, 44, 47, 48, 54, 58).

##### Seclusion

Seclusion was included in six studies (18, 25, 31, 32, 38, 78). Four of them report of seclusion together with other coercive measures, especially restraint measures. Only two examined seclusion separately. One study reported the death of a patient in seclusion who suffocated between mattress and bed sheet (78). Mattson and Sacks (25) reported of self-injury in eight out of the 66 patients in seclusion, and as the main danger the overlooking of complications. Unfortunately, no information was provided on the non-secluded control group.

##### Forced Medication

Forced medication was explicitly labeled as such in only two studies (31, 64), but further seven studies (18, 27, 42, 63, 67, 68, 74) reported intravenous or intramuscular application of an orally available substance to patients in restraint. Forced medication was mostly documented in connection with other coercive measures (restraints), and the examined harm only related to the latter measure, except for the study by Nielssen et al. (27).

#### Populations

Thirty studies (18, 21, 22, 25, 27, 29, 31, 32, 38–43, 51, 57, 58, 60–62, 64, 65, 67, 68, 70–72, 74, 78, 80) reported coercive measures in patients with schizophrenia or psychosis, 18 studies (22, 26, 29, 30, 32, 34, 38, 40, 43, 45, 46, 49, 53, 56, 69, 75–77) included dementia in their population, and affective disorders (mostly manic episodes) were diagnoses in 15 studies (16, 20–22, 25, 27, 29, 31, 32, 38, 39, 41, 49, 51, 79). States of excitation and excited delirium (ED) with no direct correlate to the ICD-10 were mentioned in 12 studies (17, 19, 33, 44, 47, 48, 52, 54, 59, 63, 66, 73), substance abuse disorders in 7 studies (21, 22, 27, 31, 38, 58, 73), delirium in 6 studies (28, 29, 36, 40, 43, 45), and personality disorders in 5 studies (21, 22, 25, 27, 38). Seventeen studies were subsumed to “others” (16, 20–24, 25, 27, 30, 31, 35, 37–39, 43, 45, 55). Two studies reported experiments on healthy subjects (14, 15).

## Discussion

The aim of this systematic literature research was to provide a comprehensive overview of the existing scientific literature on physical harm due to the use of coercive measures.

The strengths of this work are the methodological stringency, that no language restrictions were made and the fact that for the first time all forms of reported physical harm due to the use of all types of coercive interventions involving mentally ill persons were aggregated.

Our search yielded 67 eligible studies, of which the only two randomized controlled trials were conducted on healthy persons and were characterized by a small number of cases. Overall, the quality of the studies found, mostly case reports and case series, is very heterogenous, differing, e.g., in the number of cases and the documentation of the coercive measures and harm. Therefore, a quantitative synthesis for meta-analyses could not be performed.

Nevertheless, the review yields some important findings. Physical restraint can lead to cardiac deterioration and even death by cardiac arrest. Other forms of harm, such as lactate acidosis and rhabdomyolysis that might have been expected, were not reported. Of the 12 studies involving patients with ED, only Strote et al. (19) and Langslow (73) mentioned these laboratory changes in individual patients, but explicitly not as a result of the coercive measure. Nevertheless, the mechanisms of increased catcholamine release through emotional stress and especially the use of cocaine deteriorating the heart are widely discussed by Michaud (33) and Pedal et al. (52).

Almost all available studies show that physical restraint in the prone position, which at first glance may seem easier and safer for staff to apply than the supine position, bears a higher risk of fatal consequences. This has already led to guideline recommendations against the prone position (8, 83). For mechanical restraint, a variety of adverse effects has been described, including death by strangulation (40, 43) or by pulmonary embolism (42, 49). A number of the most dangerous consequences (e.g., strangulation and self-injury) can be definitively prevented by 1:1 supervision as recommended in guidelines and by the European Committee for the Prevention of Torture and Inhuman or Degrading Treatment (84). Other harms, primarily VTE, but also alterations of heart function and liver function, are an inherent risk. As the careful work of Ishida et al. (22) demonstrates, venous thrombosis has to be expected in about 1 of 10 patients even under prophylactic measures, increasing with time of exposure. Even if based solely on secondary retrospective analyses, available results suggest that mechanical restraint can increase the probability of subsequent falls in elderly patients (35, 37). Hence, prevention of falls is a rather questionable reason for the use of mechanical restraint. Regarding VTE and some other types of harm, results are somewhat inconsistent, insofar some studies are available that reported no negative effects at all. Reasons for these inconsistencies could be the different methodology, or the fact that the examination methods used were not specific or sensitive enough to detect the harm (18). Therefore, estimates of the frequency of different types of harm can be made only with caution. The available epidemiological studies suggest that relevant negative physical consequences resulting from physical restraint occur with a frequency in 1 in 100 up to 1 in 25. For other coercive measures, no estimates are possible based on the available literature. Pertaining to seclusion, there is a striking discrepancy between the widespread use of this measure and the nearly complete lack of studies on adverse events. Our literature review yielded only one older observational study with a small N (25) and one case report (78). This does not necessarily mean that seclusion is generally safe. Adverse effects of seclusion seem to be a widely underresearched topic.

### Limitations

#### Methodological Limitations

One limitation of this work is the fact that only two databases were searched. On the other hand, the additional gain from searching another database is usually estimated to be low (85, 86).

Due to the expanded search string and the fact that all study types were included, a high recall (low data loss) should be achieved. This, however, led to the discovery of a large number of irrelevant studies, so that completeness was achieved at the expense of precision. Despite the rather general and broad search string, at least one relevant article was not found: “deaths due to physical restraint” (87), while the article with the similar title “deaths due to mechanical restraint” (45) was found. The reason was that in the abstract of the unfound article only “patients” were mentioned, while our search string required a psychiatric disease in the description of the population (link with AND). For further systematic literature searches, it must therefore be considered whether the search should be extended to the full text, or whether it makes sense to extend the search string even further.

Psychological harm caused by coercive measures was not investigated. However, this should not diminish the importance of psychological consequences of coercive interventions (88, 89).

The inclusion of the 14 studies reporting on coercive measures in police custody can be critically discussed, especially as some report on additional use of weapons. However, these studies exclusively included mentally ill persons, and the mechanism of physical restraint—often in the prone position—usually does not differ from physical restraint that may be required in the context of emergency psychiatric measures (90), so that exclusion of these studies would have meant a loss of important findings on possible harm and harm mechanisms in coercive measures. The additional use of weapons such as tasers reported in some studies was not described as the cause of death.

Another downside is the heterogeneity and methodological quality of the studies and the fact that no randomized controlled trials could be included (at least concerning patients). For ethical reasons, the feasibility of RCTs on the efficacy or side effects of coercive measures on patients is very limited.

RCTs are generally not appropriate to find rare side effects, which typically are detected in large-scale observational studies or as case reports.

Due to the paucity of available RCTs and large-scale observational studies a quantitative synthesis could not be performed and frequencies of adverse events could only be roughly estimated.

#### Limitations of the Results

None of the studies found recorded all types of harm in all forms of coercive measures. In most cases, only one type of harm was investigated with only one or a few different coercive measures. The fact that only one adverse event (e.g., falls) was investigated does not mean that other harm (e.g., skin abrasions) did not occur. A direct comparison (probability of occurrence) of the several types of harm is therefore not possible. Furthermore, for some types of harm (e.g., cardiac effects) causality is difficult to determine and overlap with effects of agitation, intoxication, and administered drugs is probable.

Especially in the case of increased mortality in patients who were subjected to coercive measures, causality is difficult to determine because the patients who received coercive measures were often described as more critically ill.

Generally, a considerable reporting bias (91) has to be assumed. No studies were found that were based on interviews with patients. Only one older study (26) prospectively recorded negative effects in a cohort of patients subjected to mechanical restraint. The remainder was based on charts or reports by staff. This reporting bias probably leads to an underestimation of physical harm, especially minor harm.

Though many case series and case reports have been published, the picture of possible negative and fatal consequences is probably far from complete. For example, we found no case reports about patients who died burning themselves in mechanical restraint, whereas such accidents have been reported in newspapers (92). Also, many practitioners have anecdotal knowledge of fractures during seclusion though no such case has been published. The striking lack of observational studies and case reports on harmful events during seclusion could wrongfully lead to the assumption that seclusion is generally safe. However, evidence is missing in this area.

On the other hand, we also have only anecdotal evidence on harm being caused by abstaining from coercive interventions. This is less a medical but rather a legal and ethical issue. Freedom-restrictive coercive interventions are not a therapeutic concept of psychiatry. From a legal perspective, they are primarily safety measures imposed to prevent harm to the patient himself or others due to his behavior. The reasons to use such kind of interventions cannot be investigated in randomized controlled trials. Either a control group with no intervention would be exposed to inacceptable risks, or, if that would not be true, the intervention would not be justified. The use of coercive interventions cannot to be justified by “evidence” from studies, as has been wrongly claimed (93). Similarly, it does not make sense to question the “efficacy” of these forms of interventions, since their primary purpose is not to improve symptoms but to prevent the patients and others from danger. However, one can compare different kinds of interventions with respect to their safety for both patients and staff, and their short-term and long-term psychological effects (9).

### Conclusion and Practical Implications

Coercive interventions can cause a wide variety of somatic harm with even fatal consequences. Part of them, particularly strangulations by belts or bedrails, can be avoided by continuous 1:1 monitoring. Therefore, continuous personal supervision during such measures is necessary not only for psychological reasons but also for reasons of safety. Physical restraint in prone position should be avoided. Pulmonary thromboembolism is an inherent risk of mechanical restraint, which cannot be completely prevented by prophylactic measures. Immobilizing restraint interventions should therefore be applied for as short as possible. Further research is necessary, particularly in two areas. Large-scale prospective observational studies should assess all harmful events during coercive measures to receive robust estimates of risk ratios. This research should imperatively encompass seclusion which is completely under-researched with respect to harmful events.

In addition to further research and establishment of measures to reduce coercive interventions, the aim should be to establish a (mandatory) central register of all coercive measures, as this is the only way that statistically valid data can be recorded at all.

Concerning systematic literature searches such as this, in the next step an extension of the search strategy would have to be carried out in the next steps.

Overall, coercive measures will probably have to remain the last resort; in individual cases with highly aroused patients who represent an acute danger for themselves or others, however, the mildest means should be selected after exhausting all other measures, if the expected benefit outweighs the possible harm, and in the awareness that coercive measures can lead to significant harm and even death.

## Author Contributions

TS and XK contributed to the conception of the review. SH created the search string. XK analyzed the data and wrote the first draft of the manuscript. All authors contributed to manuscript revision and read and approved the submitted version.

## Conflict of Interest Statement

The authors declare that the research was conducted in the absence of any commercial or financial relationships that could be construed as a potential conflict of interest.
